# Comparison of three equations for estimating low‐density lipoprotein‐cholesterol in the rural northeastern region of Thailand

**DOI:** 10.1002/jcla.23429

**Published:** 2020-06-30

**Authors:** Sirawich Sonsok, Pongdech Sarakarn, Pattara Sanchaisuriya

**Affiliations:** ^1^ Department of Medical Technology Nadun Hospital Mahasarakham Thailand; ^2^ ASEAN Cancer Epidemiology and Prevention Research Group (ACEP) Faculty of Public Health Khon Kaen University Khon Kaen Thailand; ^3^ Department of Epidemiology and Biostatistics Faculty of Public Health Khon Kaen University Khon Kaen Thailand; ^4^ Department of Public Health Administration Health Promotion Nutrition Faculty of Public Health Khon Kaen University Khon Kaen Thailand

**Keywords:** agreement, cardiovascular disease, LDL‐C equation, northeastern Thailand, rural area

## Abstract

**Background:**

Cardiovascular disease is the most common cause of death worldwide, and the detection of LDL‐C contributes to reducing risks. However, the LDL‐C is rarely evaluated according to the gold standard method because it is costly and time‐consuming. This study aimed to determine the agreement of LDL‐C among three equations, namely Friedewald's equation, Puavilai's equation, and Dansethakul's equation.

**Methods:**

A cross‐sectional descriptive study.

**Results:**

Using the data of lipid measurement from a specific group of people in the remote rural area, we found that the Thai equations have more superior agreement with direct measurement than the Friedewald equation (ICC = 0.870, 95% CI = 0.857‐0.882) when the agreement of continuous data was used for total analysis. Although the categorical analysis that gave better agreement was from Friedewald equation (K index = 0.730, 95% CI = 0.720‐0.751), the findings from this study confirmed the population‐specific use of Pauvilai's equation and Dansethakul's equation for determining the LDL‐C.

**Conclusion:**

Pauvilai's equation showed better agreement with direct measurement for LDL‐C. Thus, it could be considered as an alternative for the direct method, particularly in laboratories in rural areas in Thailand.

## 1 INTRODUCTION

Cardiovascular disease (CVD) is the most common cause of death worldwide, with 17.9 million people dying from CVD in 2016.^1^ In 2012‐2015, the trends in mortality rate of CVDs in Thailand showed that increasing percentages of patients died from coronary heart disease and stroke.^2^ Lifestyle changes influence the development of CVDs.^3^ Particularly, unhealthy lifestyle habits such as high fat intake, lack of physical activity, excess alcohol consumption, and smoking are directly related to dyslipidemia.^4^, ^5^, ^6^ Ultracentrifugation is a standard method^7^ for testing both lipid and lipoprotein levels, but it is time consuming, costly, and requires sophisticated equipment.^8^, ^9^ Thus, its application is limited, particularly in countries with limited economic resources including Thailand.^10^ Previous studies showed that both lipid and lipoprotein levels can be estimated using the Friedewald equation^11^ However, triglycerides (TG) over 400 mg/dL cannot be used in this method, and underestimation compared to direct measurements has been reported when the limitation was violated.^12^, ^13^, ^14^ Recently, several equations based on various concepts and statistical methods have been reported. Some variables such as total cholesterol (TC), TG, and high‐density lipoprotein‐cholesterol (HDL‐C) were included to develop appropriate formulas involving multiple linear regression, pace regression, and mathematical modeling.^15^, ^16^, ^17^ Nevertheless, most equations were developed for a target population based on specific areas, such as those identified to have higher rates of disease. For instance, the incidence of atherogenic dyslipidemia (AD) has been reported to be higher in the urban population. Further, significant differences in lipid levels and the prevalence of dyslipidemia have been reported between urban and rural areas.^18^, ^19^

In Thailand, two equations have been proposed: One equation was developed by modifying the Friedewald method, and the other equation was developed by using pace regression.^17^ In the modified Friedewald, the denominator is changed from five to six.^20^ While both equations were validated in using similar data (ie lipid levels from Thai people), we noted that both equations were focused on hospital data of Thai people who were mostly living in the city or urban areas. The applicability of these methods was not validated in rural residents. Thus, this study aimed to evaluate the consistency and applicability of these methods for rural area residents, particularly those in the northeastern region in Thailand. Toward this goal, we determined the agreement of LDL‐C among three equations, namely Friedewald's equation, Puavilai's equation, and Dansethakul's equation in Thai people living in Nadun District.

## 2 MATERIALS AND METHODS

### 2.1 Study design

This was a descriptive cross‐sectional study that evaluated the blood and lipid profile test results of residents of Nadun district who received medical care in Nadun Hospital, Maha Sarakham Province, Thailand, between 2011 and 2017. This study was approved by the ethical committee of Khon Kaen University under the category “expedited review” status (HE612229). The study involved analysis of existing data from the hospital information system, and all data were anonymized before analysis. Thus, the need for informed consent was waived.

### 2.2 Study population

Patients who have undergone lipid profile tests and whose results were recorded in the hospital information system were evaluated. The inclusion criterion was complete laboratory results for each of the four parameters needed for the direct measurement method: TC, TG, HDL‐C, and LDL‐C, irrespective of their sex and age. In total, data from 1503 patients were included in the analysis.

### 2.3 Method

The direct detection of lipids and lipoprotein was performed using Dirui CS‐400 clinical chemistry analyzer (Dirui Industrial Co., Ltd). TC, TG, HDL‐C, and LDL‐C were measured using the cholesterol fluid monoreagent enzymatic colorimetric test; CHOD and PAP, triglyceride fluid monoreagent enzymatic colorimetric test; GPO‐PAP and HDL‐C, homogenous direct enzymatic assay; and LDL‐C, homogenous direct enzymatic assay. All reagents used were from Centronic GmbH. We used the existing data from direct measurement method to calculate the LDL‐C in each equation, with LDL‐C used as the measure to determine method agreements. Two equations from Thai developers and one equation from Friedewald were used for comparison as follows:

1. Puavilai LDL‐C (mg/dL) = TC − HDL‐C − (TG/6)
2. Dansethakul LDL‐C (mg/dL) = 0.9955 TC − 0.9853 HDL‐C − 0.1998TG + 7.1449
3. Friedewald LDL‐C (mg/dL) = TC − HDL‐C − (TG/5)

**Figure 1:**
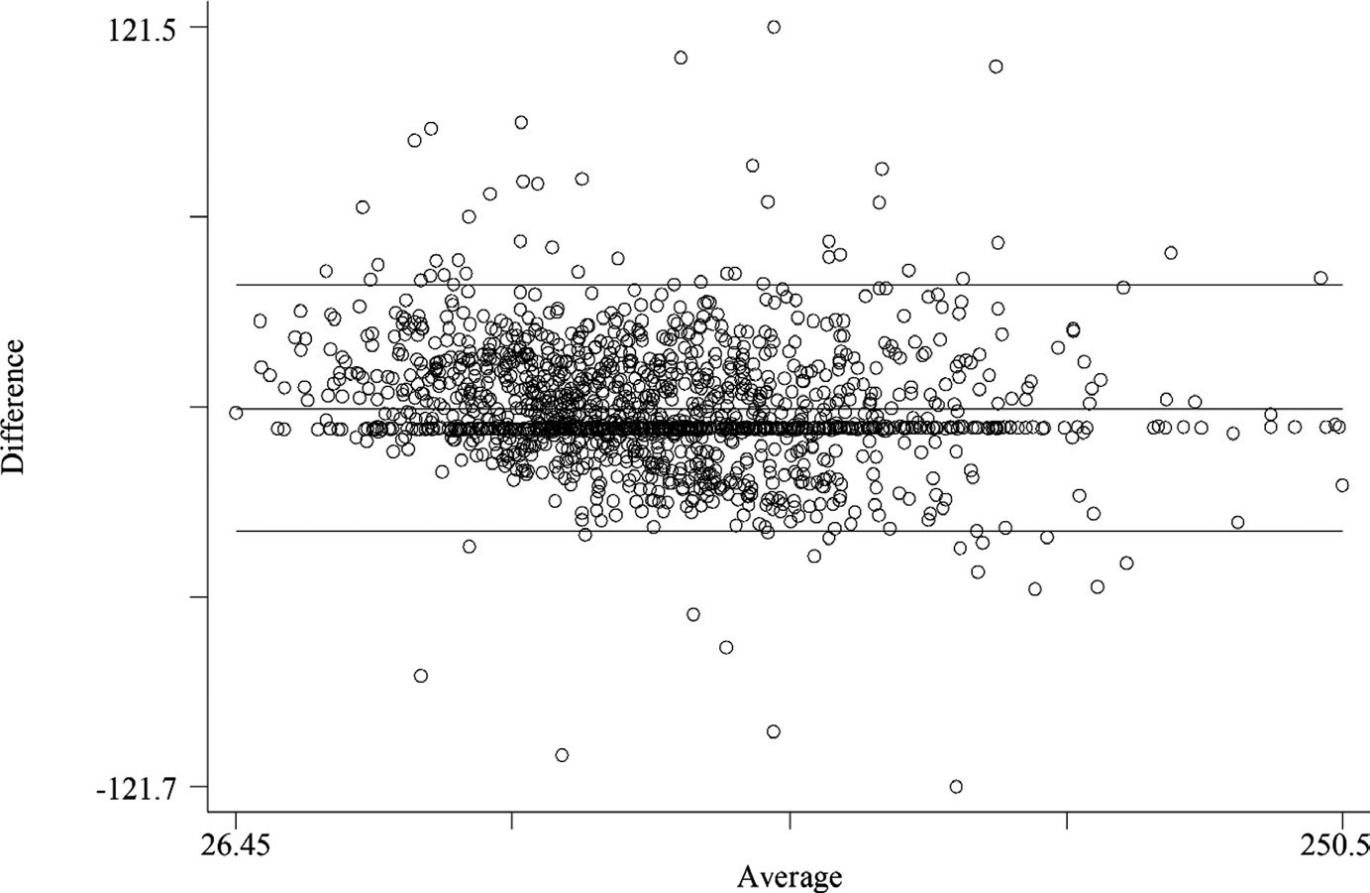
Bland‐Altman plots between enzymatic measurement of LDL‐C and the Friedewald estimated LDL‐C

When we needed to convert lipid parameters into mmol/L, we divided cholesterol by 38.67 and triglycerides by 88.57 and converted it back to mg/dL using the opposite mathematical method (Figure 1).

### 2.4 Statistical analysis

2.4

The patients’ general characteristics were presented as descriptive statistics, including frequency, percentages, range, mean or median, interquartile range (IQR), and standard deviation (SD), based on the types and distributions of data. According to the direct measurements of TC, TG, and HDL‐C were used to estimate the LDL‐C. Continuous LDL‐C was compared using the Bland‐Altman plot and intraclass correlation (ICC), while categorical LDL‐C was classified into five groups, namely <2.59, 2.59‐3.34, 3.35‐4.11, 4.12‐4.89, and ≥4.89 mmol/L based on the Thai guideline of internal medicine using only the weighted Kappa index (K index). Agreement according to the ICC was defined as good and moderate when the ICC was >0.75 and 0.5‐0.75, respectively.^21^ For agreement according to weighted Kappa index, substantial and moderate agreement were defined as a K index of 0.61‐0.80 and 0.41‐0.60, respectively.^22^ All statistical analysis were performed using STATA 15.0 software (StataCorp LLC).

## 3 RESULTS

Of the 1,503 patients, 1102 (73.3%) were women, and most subjects were aged 50‐65 years ( $\bar{X}$ = 58.8, SD = 11.4). The patients’ general characteristics are shown in Table 1. The most common diagnosis was diabetes mellitus (52.7%), followed by hypertension (30.9%), and other diseases (16.4%), including thyroid diseases, stroke, myocardial infarction, cancer, atypical headache, disease of the immune system, chronic kidney disease, and liver disease.

**Table 1:** Patient characteristics

Variables	n	%
Sex		
Male	401	26.7
Female	1102	73.3
Age, years		
<55	511	34.0
55‐65	540	35.9
≥66	452	30.1
Diagnosis		
Diabetes mellitus	792	52.7
Hypertension	465	30.9
Others	246	16.4
TC level, mmol/L		
≤3.88	181	12.1
3.89‐5.17	686	45.6
5.18‐6.46	478	31.8
>6.46	158	10.5
TG level, mmol/L		
≤1.69	512	34.1
1.70‐2.25	338	22.5
2.26‐5.63	590	39.3
>5.63	63	4.1

	Mean (SD)	Median (IQR)	Range
Age (y)	58.8 (11.4)	59 (16)	20‐92
Total cholesterol (mmol/L)	5.08 (1.09)	5.02 (1.34)	1.60‐10.24
Triglycerides (mmol/L)	2.47 (1.43)	2.08 (1.46)	0.41‐10.16
HDL‐C (mmol/L)	1.21 (0.37)	1.18 (0.48)	0.27‐2.53
LDL‐C direct method (mmol/L)	2.91 (0.91)	2.79 (1.15)	0.54‐6.89
LDL‐C Friedewald equation (mmol/L)	2.74 (1.00)	2.66 (1.29)	0.28‐6.65
LDL‐C Puavilai equation (mmol/L)	2.93 (0.98)	2.84 (1.26)	0.49‐6.79
LDL‐C Dansethakul equation (mmol/L)	2.92 (1.00)	2.84 (1.27)	0.46‐6.80

**Figure 2:**
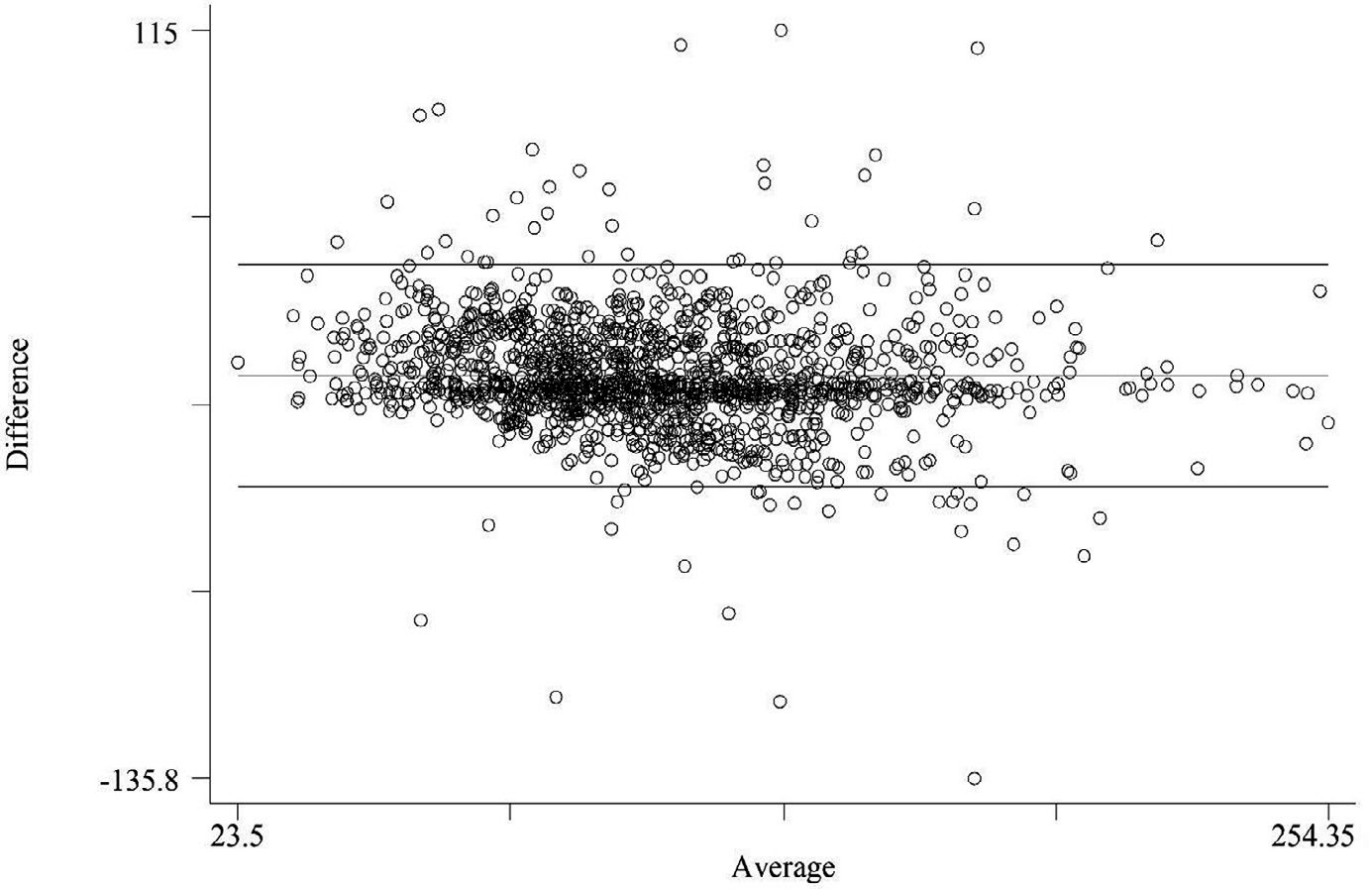
Bland‐Altman plots between enzymatic measurement of LDL‐C and the Puavilai estimated LDL‐C

For the analysis of LDL‐C agreement, the ICC (95% CI) was 0.870 (0.857‐0.882), 0.858 (0.844‐0.871), and 0.858 (0.844‐0.870) for Puavilai's equation, Dansethakul's equation, and the modified Friedewald equation, respectively (Figure 2). Subgroup analysis of the three methods for TG found highest agreement at TG ≤ 1.69 mmol/L. The ICCs (95% CI) were 0.946 (0.936‐0.955), 0.946 (0.935‐0.954), and 0.945 (0.935‐0.954), respectively. For TC, we found highest agreement at TC > 6.46 mmol/L. The ICCs (95% CI) were 0.834 (0.773‐0.879), 0.827 (0.793‐0.855), and 0.826 (0.792‐0.854), respectively. For age groups, we found highest agreement at age >65 years. The ICCs (95% CI) were 0.879 (0.856‐0.898), 0.872 (0.848‐0.892), and 0.872 (0.848‐0.892), respectively. In women, the ICCs (95% CI) were 0.870 (0.855‐0.884), 0.859 (0.843‐0.874), and 0.859 (0.843‐0.874), respectively. For the subjects with diabetes, we found ICCs (95% CI) of 0.881 (0.864‐0.895), 0.870 (0.852‐0.886), and 0.870 (0.852‐0.886), respectively.

**Figure 3:**
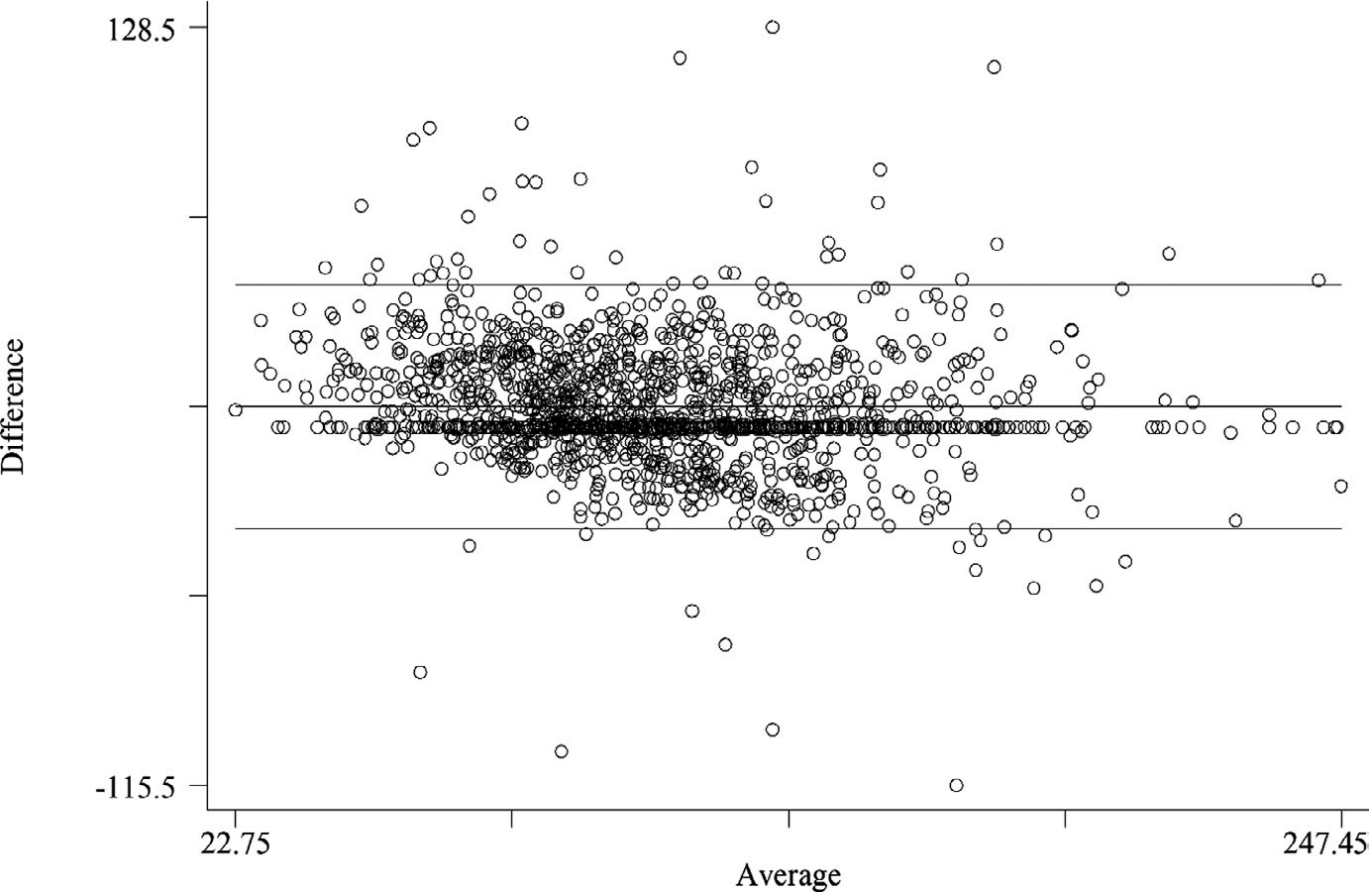
Bland‐Altman plots between enzymatic measurement of LDL‐C and Dansethakul estimated LDL‐C

For categorical agreement according to the K index, the modified Friedewald equation showed the highest K index (95% CI) at 0.730 (0.720‐0.751), followed by Puavilai's equation and Dansethakul's equation at 0.708 (0.692‐0.712) and 0.693 (0.688‐0.697), respectively (Figure 3). Subgroup analysis showed the highest agreement when TG was <1.69 mmol/L for Friedewald LDL‐C and Puavilai LDL‐C, with a K index (95% CI) of 0.759 (0.716‐ 0.780) and 0.739 (0.729‐0.755), respectively. However, for Dansethakul LDL‐C, the highest agreement is from TG 1.70‐2.25 mmol/L, with a K index (95% CI) of 0.727 (0.687‐0.755). All equations yielded the highest agreement for TC of 3.89‐5.17 mmol/L, with a K index (95% CI) of 0.581 (0.553‐0.610), 0.541 (0.476‐0.559), and 0.534 (0.497‐0.544) for Friedewald LDL‐C, Puavilai LDL‐C, and Dansethakul LDL‐C, respectively. With respect to age group, Friedewald LDL‐C and Dansethakul LDL‐C showed the highest agreement in age over 65 years, while Puavilai LDL‐C showed the high agreement in age 55‐65 years. The K indices (95% CI) were 0.754 (0.734‐0.814), 0.718 (0.689‐0.735), and 0.708 (0.674‐0.715) for Friedewald, Puavilai, and Dansethakul LDL‐C, respectively. Among the female subjects, Friedewald LDL‐C showed the highest agreement, with a K index (95% CI) of 0.731 (0.705‐0.760). Both Puavilai and Dansethakul LDL‐C showed the highest agreement for men, with a K index of 0.708 (0.686‐0.729), and 0.691 (0.658‐0.701), respectively. For analysis according to diagnosis, superior agreement of Friedewald and Puavilai LDL‐C was found among subjects with diabetes. The K indices (95% CI) were 0.765 (0.716‐0.781) and 0.727 (0.708‐0.742), respectively, while the Dansethakul LDL‐C showed the best agreement among subjects diagnosed with other diseases. The K index (95% CI) was 0.721 (0.671‐0.747).

The results of the Bland‐Altman analysis showed that the mean differences between the direct method LDL‐C and the Friedewald LDL‐C were 0.167 (95% CI, 0.141‐0.193), −0.022 (95% CI, −0.046‐0.002) for Puavilai LDL‐C, and −0.014 (95% CI, −0.040‐0.012) for Dansethakul LDL‐C.

## 4 DISCUSSION

This study investigated the agreement in LDL‐C between the direct method and three equations. We found that the Puavilai equation showed superior agreement over the other two equations, with a high ICC not only in the overall analysis, but also in the subgroup analysis of women, subjects with diabetes, and subjects with hypertension (Table 2). The higher ICCs may be because the subjects recruited were Thai villagers, similar to the population used to develop the Puavilai equation and different from that in the Dansethakul and Friedewald equations that mainly included urban residents. Moreover, the Puavilai equation had been proposed under the assumption of the lower “very low‐density lipoprotein cholesterol:TG ratio” than the Friedewald study, which resulted in a comparable calculated LDL‐C that is closer to the LDL‐C measured using the direct method.^20^ The similar target population also resulted in similar lipid levels between the current study and the Puavilai study. This effect can be observed from the higher ICCs. Meanwhile, when agreement was analyzed according to the K index, the modified Friedewald equation showed the highest agreement in the overall analysis and the subgroup of patients with TG below 150 mg/dL, aged over 65 years, female subjects, and with diabetes (Table 3). However, there could be differences in findings between two statistical methods based on scale measurements, as reported previously.^23^ The high K index of the Friedewald equation can be attributed to the definition of the study population. First, for hospital population‐based studies, although we found a higher K index of the Friedewald equation (K = 0.730, 95% CI, 0.720‐0.751), it is still lower than that in Rim's study^15^ based on US guidelines (K = 0.856). The difference could be due to the difference in characteristics between the study population, including race and dietary patterns.^24^, ^25^ Second, for general population‐based studies, although the population was not covered in our study, Rim's study showed that the K index of Friedewald's equation was lower than that in the hospital population‐based study (K = 0.804). The difference could be due to variations in patient characteristics, including ethnicity and geographic location.^26^ The Bland‐Altman analysis showed that the highest mean difference in LDL‐C was between the direct method and the Friedewald method, indicating that this method had the poorest agreement or has more bias to estimate the LDL‐C. This could be possibly due to the differences in race and characteristics of the applied method of measurement,^27^ as mentioned above.

**Table 2:** Quantitative agreement of LDL‐C between enzymatic measurement and the three equations according to the intraclass correlation coefficient

Friedewald				Puavilai				Dansethakul			
	n	ICC	95% CI		n	ICC	95% CI		n	ICC	95% CI
All	1503	0.858	0.844‐0.870	All	1503	0.870	0.857‐0.882	All	1503	0.858	0.844‐0.871
TG ≤ 1.69 mmol/L	512	0.945	0.935‐0.954	TG ≤ 1.69 mmol/L	512	0.946	0.936‐0.955	TG ≤ 1.69 mmol/L	512	0.946	0.935‐0.954
TG 1.70‐2.25 mmol/L	338	0.936	0.921‐0.948	TG 1.70‐2.25 mmol/L	338	0.936	0.921‐0.949	TG 1.70‐2.25 mmol/L	338	0.936	0.921‐0.949
TG 2.26‐5.63 mmol/L	590	0.918	0.904‐0.930	TG 2.26‐5.63 mmol/L	590	0.922	0.908‐0.934	TG 2.26‐5.63 mmol/L	590	0.918	0.904‐0.931
TG > 5.63 mmol/L	63	0.880	0.801‐0.927	TG > 5.63 mmol/L	63	0.885	0.809‐0.930	TG > 5.63 mmol/L	63	0.880	0.802‐0.927
TC 5.18‐6.46 mmol/L	478	0.827	0.793‐0.855	TC > 6.46 mmol/L	478	0.834	0.773‐0.879	TC 5.18‐6.46 mmol/L	478	0.826	0.792‐0.854
TC > 6.46 mmol/L	158	0.823	0.758‐0.871	TC 5.18‐6.46 mmol/L	478	0.832	0.799‐0.860	TC > 6.46 mmol/L	158	0.822	0.757‐0.870
TC 3.89‐5.17 mmol/L	686	0.821	0.792‐0.846	TC 3.89‐5.17 mmol/L	686	0.830	0.803‐0.854	TC 3.89‐5.17 mmol/L	686	0.821	0.792‐0.846
TC ≤ 3.88 mmol/L	181	0.635	0.510‐0.727	TC ≤ 3.88 mmol/L	181	0.632	0.507‐0.726	TC ≤ 3.88 mmol/L	181	0.636	0.512‐0.728
Age >65 y	452	0.872	0.848‐0.892	Age >65 y	452	0.879	0.856‐0.898	Age >65 y	452	0.872	0.848‐0.892
Age <55 y	511	0.858	0.834‐0.879	Age <55 y	511	0.877	0.855‐0.895	Age <55 y	511	0.858	0.833‐0.879
Age 55‐65 y	540	0.848	0.823‐0.870	Age 55‐65 y	540	0.859	0.835‐0.879	Age 55‐65 y	540	0.848	0.823‐0.870
Female sex	1102	0.859	0.843‐0.874	Female sex	1102	0.870	0.855‐0.884	Female sex	1102	0.859	0.843‐0.874
Male sex	401	0.847	0.817‐0.873	Male sex	401	0.863	0.836‐0.886	Male sex	401	0.847	0.817‐0.873
Diabetes	792	0.870	0.852‐0.886	Diabetes	792	0.881	0.864‐0.895	Diabetes	792	0.870	0.852‐0.886
Other diseases	246	0.846	0.806‐0.878	Other diseases	246	0.865	0.830‐0.894	Other diseases	246	0.845	0.805‐0.877
Hypertension	465	0.839	0.810‐0.864	Hypertension	465	0.851	0.824‐0.875	Hypertension	465	0.840	0.811‐0.865

**Table 3:** Weighted Kappa index between enzymatic measurement LDL‐C and the three equations for estimating LDL‐C

Friedewald				Puavilai				Dansethakul			
	n	K	95% CI		n	K	95% CI		n	K	95% CI
All	1503	0.730	0.720‐0.751	All	1503	0.708	0.692‐0.712	All	1503	0.692	0.688‐0.697
TG ≤ 1.69 mmol/L	512	0.759	0.716‐0.780	TG ≤ 1.69 mmol/L	512	0.739	0.729‐0.755	TG 1.70‐2.25 mmol/L	338	0.727	0.687‐0.755
TG 1.70‐2.25 mmol/L	338	0.747	0.730‐0.790	TG 1.70‐2.25 mmol/L	338	0.735	0.701‐0.771	TG ≤ 1.69 mmol/L	512	0.700	0.663‐0.726
TG 2.26‐5.63 mmol/L	590	0.697	0.665‐0.718	TG 2.26‐5.63 mmol/L	590	0.669	0.648‐0.715	TG 2.26‐5.63 mmol/L	590	0.664	0.649‐0.685
TG > 5.63 mmol/L	63	0.531	0.460‐0.599	TG > 5.63 mmol/L	63	0.664	0.597‐0.705	TG > 5.63 mmol/L	63	0.548	0.439‐0.651
TC 3.89‐5.17 mmol/L	686	0.581	0.553‐0.610	TC 3.89‐5.17 mmol/L	686	0.541	0.476‐0.559	TC 3.89‐5.17 mmol/L	686	0.534	0.497‐0.544
TC 5.18‐6.46 mmol/L	478	0.564	0.524‐0.599	TC 5.18‐6.46 mmol/L	478	0.539	0.533‐0.577	TC 5.18‐6.46 mmol/L	478	0.524	0.481‐0.531
TC > 6.46 mmol/L	158	0.530	0.435‐0.533	TC > 6.46 mmol/L	158	0.494	0.412‐0.552	TC > 6.46 mmol/L	158	0.456	0.402‐0.521
TC ≤ 3.88 mmol/L	181	0.427	0.381‐0.453	TC ≤ 3.88 mmol/L	181	0.467	0.396‐0.481	TC ≤ 3.88 mmol/L	181	0.451	0.399‐0.470
Age >65 y	452	0.754	0.734‐0.814	Age 55‐65 y	540	0.718	0.689‐0.735	Age >65 y	452	0.708	0.674‐0.715
Age <55 y	511	0.739	0.718‐0.752	Age >65 y	452	0.706	0.681‐0.742	Age <55 y	511	0.693	0.664‐0.719
Age 55‐65 y	540	0.702	0.683‐0.703	Age <55 y	511	0.698	0.638‐0.712	Age 55‐65 y	540	0.678	0.660‐0.726
Female sex	1102	0.731	0.705‐0.760	Male sex	401	0.708	0.686‐0.729	Male sex	401	0.691	0.658‐0.701
Male sex	401	0.718	0.679‐0.733	Female sex	1102	0.704	0.690‐0.710	Female sex	1102	0.688	0.676‐0.713
Diabetes	792	0.765	0.716‐0.781	Diabetes	792	0.727	0.708‐0.742	Other diseases	246	0.721	0.671‐0.747
Hypertension	465	0.711	0.694‐0.738	Other diseases	246	0.683	0.668‐0.709	Diabetes	792	0.702	0.668‐0.705
Other diseases	246	0.646	0.603‐0.691	Hypertension	465	0.681	0.659‐0.702	Hypertension	465	0.653	0.650‐0.666

This study has two limitations. First, time might affect lifestyle, dietary patterns, and the development of medical technology, but time effect was not considered in the analysis. For example, a different period of time would have found a different incidence of dyslipidemia.^28^, ^29^ Second, unlike most previous studies that used ultracentrifugation, we used the direct enzymatic method to measure the LDL‐C value set as the reference. Despite these limitations, our study remains valuable because to our best knowledge, this is the first study to compare the different methods of estimating LDL‐C concentration in Thailand, particularly in northeastern Thailand where most people have been reported to be at higher risk of higher levels of LDL‐C and TG and lower level of HDL‐C.^30^ Further studies are needed to evaluate the reliability of various equations in specific disease groups in Thailand.

In conclusion, compared to Friedewald's and Dansethakul's equation, the Puavilai equation showed better agreement with direct measurement for LDL‐C. Thus, it could be considered as an alternative method to the direct method for measuring LDL‐C, particularly in laboratories in rural areas.
